# Supplementary material for: Leader emergence and affective empathy: A dynamic test of the dual-hormone hypothesis
Source: PLoS One. 2020 Dec 30;15(12):e0244548. doi: 10.1371/journal.pone.0244548 (PMC7773240; doi:10.1371/journal.pone.0244548)
Supplement: Attachment — Submitted filename: Response to Reviewers.docx [file pone.0244548.s001.docx]

**Response to Reviewers**

Kindly note that our responses are included in red after each of the Reviewer’s italicized comments.

*My applause to the authors of the PONE-D-20-32353! The model tested is amazingly relevant in these trying times in which we witness leadership of all forms tested under high stress.* Thank you for taking the time to review our work. We very much appreciated your insightful comments and your praise.

1. *The design is clear and relevant both in the research world and in practice.* Thank you.
2. *The measures chosen are quite appropriate and their processing was handled well.* Thank you. The PSE coding was a laborious task that required much attention to detail.
3. *The data analysis was meticulous and explained well.* Thank you.
4. *The results offer another important step in understanding the psycho-physiology of leadership.* Thank you.
5. *The choice of personalized power as the key motivational variable is fine for this arousal (experimental) design. Being a part of the original research, it was socialized power that was thought and found to be key to effective leadership. But in this design, the arousal was a perceived zero-sum competition. In such a situation, personalized power would be both the fastest to be aroused and most immediately relevant. A comment about that should be made in the Discussion. That would clarify a possible confusion in thinking about emergent leadership in highly competitive settings versus effective leadership.* We value this comment and acknowledge that it is important to address this distinction between emergent leadership, where p Power may be particularly useful, and effective leadership in organizations where s Power is key, as your pioneering work has long established. This will clarify any potential confusion on our choice for using one ‘face of power’ of the other. We include this new section in our revised manuscript on page 30 (i.e., the entire second paragraph immediately following the “Discussion” heading).
6. *The endocrinological hypotheses are sound. But there is something more subtle possibly involved. Testosterone has been linked primarily to instrumental behavior. Often, through its constituent role in creating vasopressin, the instrumental causality becomes clear. At modest doses, vasopressin is also linked to Parasympathetic Nervous System arousal which ameliorates the negative effects of stress. Since cortisol is a clear endocrine marker of stress, the interactions found in this study make sense. But at higher doses, both testosterone and vasopressin can drive more, in McClelland’s terms, “power stress” with its effect of increasing stress hormones, like cortisol. This is no way threatens the validity or importance of the findings in this study, but it suggests a dosage effect worthy of comment in the Discussion to avoid simplistic conclusions about T and C and leadership effectiveness and sustainability.* This was also a notable comment and we agree that we should have better articulated how complex these hormonal mechanisms truly are. In the revised manuscript, we allude to vasopressin (arginine vasopressin or AVP) and its effect on both T and C. This section is found on page 34, added at the end of the first paragraph following the heading “Limitations and future research.” We are also aware of McClelland’s research on the relationship between AVP and the need for achievement (nAch) but opted to leave this detail out of the manuscript because our study does not address implicit motives other than power.
